# PLGA-PEI nanoparticle covered with poly(I:C) for personalised cancer immunotherapy

**DOI:** 10.1007/s13346-024-01557-2

**Published:** 2024-03-01

**Authors:** Lorena Gonzalez-Melero, Edorta Santos-Vizcaino, Ruben Varela-Calvino, Iria Gomez-Tourino, Aintzane Asumendi, Maria Dolores Boyano, Manoli Igartua, Rosa Maria Hernandez

**Affiliations:** 1 NanoBioCel Research Group, Laboratory of Pharmaceutics, School of Pharmacy, University of the Basque Country (UPV/EHU), Vitoria-Gasteiz, Spain; 2 Bioaraba, NanoBioCel Research Group, Vitoria-Gasteiz, Spain; 3 Biomedical Research Networking Centre in Bioengineering, Biomaterials and Nanomedicine (CIBER-BBN), Institute of Health Carlos III, Madrid, Spain; 4 Department of Biochemistry and Molecular Biology, School of Pharmacy, University of Santiago de Compostela, Santiago, Spain; 5 Centre for Research in Molecular Medicine and Chronic Diseases (CiMUS), University of Santiago de Compostela, Santiago, Spain; 6 Health Research Institute of Santiago de Compostela (IDIS), Santiago, Spain; 7 Biocruces Bizkaia Health Research Institute, 48903 Barakaldo, Spain; 8 Department of Cell Biology and Histology, Faculty of Medicine and Nursing, University of the Basque Country (UPV/EHU), 48940 Leioa, Spain

**Keywords:** Cancer, Immunotherapy, Nanoparticle, Neoantigen, PLGA, Poly(I:C), Vaccine

## Abstract

Melanoma is the main cause of death among skin cancers and its incidence worldwide has been experiencing an appalling increase. However, traditional treatments lack effectiveness in advanced or metastatic patients. Immunotherapy, meanwhile, has been shown to be an effective treatment option, but the rate of cancers responding remains far from ideal. Here we have developed a personalized neoantigen peptide-based cancer vaccine by encapsulating patient derived melanoma neoantigens in polyethylenimine (PEI)-functionalised poly(lactic-co-glycolic acid) (PLGA) nanoparticles (NPs) and coating them with polyinosinic:polycytidylic acid (poly(I:C)). We found that PLGA NPs can be effectively modified to be coated with the immunoadjuvant poly(I:C), as well as to encapsulate neoantigens. In addition, we found that both dendritic cells (DCs) and lymphocytes were effectively stimulated. Moreover, the developed NP was found to have a better immune activation profile than NP without poly(I:C) or without antigen. Our results demonstrate that the developed vaccine has a high capacity to activate the immune system, efficiently maturing DCs to present the antigen of choice and promoting the activity of lymphocytes to exert their cytotoxic function. Therefore, the immune response generated is optimal and specific for the elimination of melanoma tumour cells.

**Graphical abstract:**

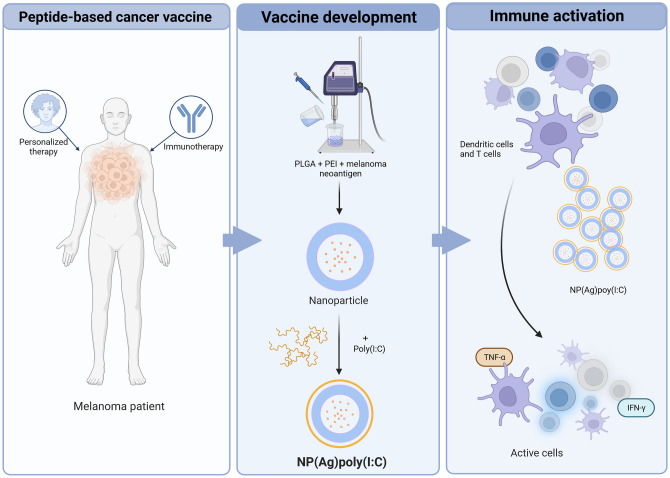

Created with BioRender.com

**Supplementary Information:**

The online version contains supplementary material available at 10.1007/s13346-024-01557-2.

## Introduction

Melanoma is an aggressive type of skin cancer that originates in melanocytes, the cells that produce the pigment that gives colour to the skin. Although much less common than non-melanoma skin cancers, melanoma is the leading cause of death among them [1]. In general, the incidence of melanoma has experienced an alarming increase worldwide over the past several decades, particularly in regions with fair-skinned populations and high levels of exposure to ultraviolet (UV) radiation [1, 2]. Surgical excision has always been the primary treatment for melanoma [3]. However, not all tumours are candidates for tumour resection. Fortunately, there has been a significant advance in the treatment of melanoma in recent years, which has changed the outcome of the disease [4]. Among them, immunotherapy has proven to be an effective treatment option for melanoma, even for advanced or metastatic patients [4], for which traditional treatments have failed to be successful. However, high cost, high heterogeneity among patients, and immune-related adverse effects are the limitations and challenges of current immunotherapy [5–7]. Moreover, only a few therapeutic options are available for patients with advanced-stage melanoma [8].

In immunotherapy, two categories can be differentiated based on their ability to activate the host immune system: active and passive immunotherapies [9]. Active immunotherapy focuses on generating an immune response in the patient to fight cancer cells; and in passive immunotherapy, immune molecules are administered to patients, providing immediate but short-lived protection as the patient does not retain the ability to generate these molecules by themselves [10]. Examples of passive immunotherapy include immune checkpoint inhibitors (ICIs), which re-activate the pre-existing immune response by blocking the inhibitory molecules of effector lymphocytes. Therefore, for passive immunotherapies to be effective, there must be a prior immune response in the patient's body [11], and as a result many people do not respond to treatment. In fact, the success rate varies significantly among cancers, and even in responsive cancers it is below 40% [12]. On the contrary, active immunotherapies boost the response of the immune system against cancer cells and install lasting immune protection [9].

Cancer vaccines are part of active immunotherapies and represent an exciting approach for cancer treatment. They can be classified based on their biological formulation or antigen source [13, 14]. Peptide-based cancer vaccines, as their name suggests, elicit tumour-specific T cell-derived immune responses by the administration of tumour antigens. These peptides are purified, recombinant, or synthetically engineered epitopes derived from tumour cells that can be presented by antigen-presenting cells (APCs) to activate T lymphocytes against malignant cells [15]. The peptides can be derived from tumour neoantigens, that is, the products of mutated genes specific to tumour cells [16]. These mutations are completely random and each patient has a unique mutation profile, so their presence is highly individual. Consequently, the development of peptide vaccines derived from neoantigens represents a personalised therapy. It should be noted that not all mutations give rise to a neoantigen, and not all neoantigens have the same capacity to generate an anti-tumour effect. Therefore, the neoantigens of interest are those mutations that give rise to a neoepitope that is recognised by T cells and that generate an antitumour effect [16].

The use of neoantigens as a target is considered a safe and potent approach because their presence is limited to tumour cells and the T cells that recognise them are not subjected to central immune tolerance [17]. In fact, peptide vaccines derived from neoantigens have shown potential to activate T cells, while being safe, simple to manufacture and, compared to traditional treatments, are administered with minimal toxicity [18–20]. Moreover, antigen loss and high heterogeneity of tumours can be successfully confronted by multiple neoepitope targeting [18, 21, 22]. Thus, peptide vaccines are expected to be useful even in metastatic cancers. However, the lack of efficacy of peptide antigens has been reported, as they may be degraded in the physiological environment by peptidases, or because they may be eliminated through the blood vasculature instead of through the lymphatic vessels draining the injection site [23]*.* In other words, tumour antigens often present difficulties in eliciting an effective immune response.

For effective tumour cell killing, it is essential that dendritic cells (DCs), which are highly efficient APCs, participate in the cross-presentation of specific epitopes on major histocompatibility complex class I (MHC I) molecules. This promotes the activation of CD8^+^ T-lymphocytes for direct cellular immune response [9, 22, 24]. For that aim, peptides must satisfy certain requirements, which are easily met by combining them with adjuvants [25]. Hence, in peptide vaccines, the requirements to be met by adjuvants include: protect the peptide and prevent its degradation, promote its uptake by APCs and induce the correct activation of immune cells.

When choosing adjuvants, it important to bear in mind that they can be subdivided into two main classes: antigen delivery systems, and immunostimulatory molecules. Ideally, both types of adjuvants should be formulated together with the antigen as their effect is usually synergistic [26, 27]. Delivery systems would protect and transport the peptide, like nanoparticulate carriers, that are primarily internalised by APCs [28–30]. As for the activation and maturation of APCs, optimal and strong activation can be achieved by a danger signal, or pathogen-associated molecular pattern (PAMP), via toll-like receptors (TLRs). Therefore, TLR ligands are often used as immune adjuvants [26, 31, 32].

In this context, the development of poly(lactic-co-glycolic acid) (PLGA) nanoparticles (NPs) emerges as a simple approach to improve the efficacy of peptide-based immunotherapy in the treatment of melanoma. PLGA formulations are approved for parental applications in humans by the European Medicines Agency (EMA) and by U.S. Food and Drug Administration (FDA) due to their good bioavailability, biodegradable and biocompatible structure and overall good safety profile [33]. PLGA consists of a hydrophobic polymeric backbone that can be formulated into particles with an aqueous or hydrophobic core by varying the preparation technique. Consequently, depending on the nature of the loaded substances, they become entrapped within the polymeric matrix, such as in spherical particles, incorporated into the core of capsules, or adhered to the surface of the particle. On top of that, PLGA particles can be easily modified by surface coating or chemical conjugation, which expands the repertoire of target molecules and active substances that can bind to the NPs [34, 35]. PLGA particles can be modified with several molecules, like polyethylenimine (PEI), a cationic polymer that promotes endosomal escape and positivises the surface potential of the PLGA NPs [36]. A positive PLGA NP manages to bind to cell membranes which are negatively charged, thereby promoting retention in tumour tissue or easing cellular uptake [37]. In addition, the positive groups of PEI can interact electrostatically with negatively charged nucleic acids, among others [36]. Therefore, PLGA-PEI NPs offer a number of advantages for peptide vaccination not only favouring cross-presentation, but also protecting peptides from degradation or co-delivering the peptide with an immunoadjuvant [38–40].

Polyinosinic:polycytidylic acid (poly(I:C) is a synthetic PAMP that mimics viral genomic double-stranded RNA (dsRNA), and therefore is a toll-like receptor 3 (TLR-3) agonist [41]. As such, it elicits the secretion of type I interferon (IFN) and pro-inflammatory cytokines to induce DC maturation and favour antigen cross-presentation, resulting in the induction of CD8^+^ T cells and a CD4^+^ adaptive T cell response with a Th1-type profile [42–45]. As a result, poly(I:C) has been widely used in cancer treatment [46–48]. However, their efficacy increases when administered in particulate formulations, as the effect of both types of adjuvant is synergistic [41].

In this article, we aimed to develop a poly(I:C) covered PLGA-PEI NP to deliver a personalised melanoma neoantigen and generate an effective and specific immune activation. Here, we optimised the developed NP to efficiently entrap the neoantigen, and to achieve a complete poly(I:C) coating. Next, we examined its uptake by APCs, as well as its potential toxicity, and we determined the ability of the NP to mature DCs. Finally, we stablished its ability to induce a specific response.

## Materials y methods

### Preparation of PLGA NPs

The melanoma neoantigen was custom made from ChinaPeptides. The neoantigen originating from a melanoma patient selected for the experiment as chosen based on previous work [49]. Briefly, the study identified melanoma patients' DNA mutations and their corresponding amino acid sequences. For experimental validation, six neoantigens of 15 amino acids in length were selected per patient. In this article, neoantigens corresponding to one patient were selected and synthesised into two peptides (A and B) (Table 1), both of 45 amino acids in length.

**Table 1:** Sequences of the peptides A and B

**Peptide A**	DWLEWLRQLSLELLKFRDQSLSYHHTMVVQIGRFANYFRNLLPSN
**Peptide B**	MRHSFFSEVNWQDVYRLFMHHVFLEPITCVCSRRFYQFTKLLDSV

NPs were prepared by the solvent extraction-evaporation of a double emulsion (w/o/w) method. Briefly, 80 mg of PLGA (50:50 lactide–glicolide ratio. Resomer-RG503; MW 40,600; viscosity 0.41 dl/g. Evonik, Germany) and 1.04 mg of PEI (polyethylenimine, branched form with molecular weight 25,000Da. Sigma Aldrich, Germany) were dissolved in 1.6 ml of DCM (dichloromethane) and emulsified with 80 µL of each peptide suspension in miliQ water using 30 seconds of sonication with a Branson sonifier 250 in an ice bath to avoid high temperatures. The resulting w/o emulsion was then mixed with 8 ml of 5% (w/v) PVA (polyvinyl alcohol. Sigma Aldrich, Germany) and sonicated for an additional 1 minute. Finally, the w/o/w emulsion was poured into a 16 ml 2% (v/v) isopropanol solution and stirred for 2 hours to allow for solvent evaporation. The resultant NPs were washed 3 times in miliQ water and freeze-dryed with 15% (w/w) trehalose for 42 h (Lyobeta 15, Telstar^®^).

### Poly(I:C) coating of PLGA NPs

The negatively charged poly(I:C) (polyinosinic:polycytidylic acid. Sigma Aldrich, Germany) was attached to the positive surface of the NPs by electrostatic interaction. Freeze-dryed NPs were resuspended at different concentrations, ranging 0.9–15 mg/ml, and incubated with poly(I:C) (50–350 µg/ml) at 4 °C on a rotating mixer. This test was repeated at two pHs, including neutral pH and an acidic pH of 4.6. After 3 hours, NPs were centrifuged. Supernatant was stored for poly(I:C) indirect measurement, and NPs were re-suspended in distilled water for size and Z-potential measurements.

### NP size, size distribution and Z potential determination

The mean particle size (Z-average diameter) and the polydispersity index (PDI) of the NPs were analysed by Dynamic Light Scattering (DLS) and Nanoparticle Tracking Analysis (NTA). The Z-potential was determined through Laser Doppler micro-electrophoresis. A Malvern^®^ Zetasizer NanoZS Model ZEN3600 (Malvern Instruments Ltd., UK) was used for DLS and Z-potential measurements.

NTA analysis were performed with NanoSight LM10 system (Malvern Panalytical, Malvern, UK). Brownian motion rate was measured with a fast video-capture and particle-tracking software. Each video was analysed to obtain the mean, mode, and median NP size. 3 consecutive video recording of 60 s each were taken for every sample quantified.

### Peptide encapsulation

Peptide encapsulation was determined by microBCA assay (Thermo Fisher Scientific, Massachusetts, USA). NPs were disrupted with 0.1 N NaOH at 37°C for 30 min under orbital rotation to determine encapsulation efficiency (EE%) (Eq. (1)). The same process but suspending NPs in PBS was used to determine surface absorbed protein (SAP) (Eq. (2)). After incubation, SAP was released to the PBS and the NPs were removed by centrifugation at 10,000 g for 5 min. Protein quantification by microBCA was performed in a linear working range of 0–30 μg/ml.

1 $$EE\; \left(\%\right)= \frac{Protein\; loading}{Theoretical\; protein\; loading} \times 100$$

2 $$SAP\; \left(\%\right)=\frac{Amount\; of\; protein\; in\; the\; surface}{Protein\; loading} \times 100$$

3 $$Protein\; loading\; \left({}^{w}\!\big/\!{}_{w}\;\%\right)=\frac{Amount\; of\; protein\; in\; NP}{Amount\; of\; NP} \times 100$$

### Poly(I:C) coating assessment

Poly(I:C) coating was indirectly assessed using a SimpliNano^™^ spectrophotometer (GE Healthcare, UK). Briefly, after poly(I:C) coating, NPs were centrifuged and the collected supernatant was measured by quantifying absorbance at 260 nm and calculating the 260/280 and 260/230 ratios. The detected signal was extrapolated to quantity by means of a standard line prepared with known concentrations of poly(I:C). The resulting concentration of the difference between the theoretical concentration and the detected concentration is the amount bound to the NPs.

### SEM

A Hitachi S-4800 FEG-SEM (Field Emission Gun – Scanning Electron Microscope) was used for image acquisition. Prior to observation, 5 µl of the suspended NPs were deposited on a silicon sheet and left to dry. Subsequently, the silicon sheets (5 × 5 mm) were mounted on an aluminium support using carbon adhesive tape. Once dried and mounted on the support, 10 nm of gold was deposited on the sample using an Emitech K550X ion sprayer.

### Phagocytosis of the NPs

To analyse NP phagocytosis in microscopy, NPs were stained. During NP preparation 0.5% DiD was added in the PLGA phase. The rest of NP preparation was performed without changes.

The macrophage cell line RAW 264.7 (ATCC, USA) was cultured following the manufacturer’s instructions. RAW 264.7 cells were cultured in covers, previously sterilized and placed in a 24 well plate, at a concentration of 20,000 cells/cm^2^. Cells were kept incubating overnight to ensure cell adhesion, and the following day they were cultured with dyed NPs for 24h.

Covers were gently washed with DPBS (Dulbecco’s - Phosphate Buffered Saline) and cells were fixed with 4% formaldehyde for 10 min. Then, macrophages were permeabilised with 0.1% Triton for 3–5 min, and dyed with Alexa Fluor^™^ 488 Phalloidin (1:40) (Thermo Fisher Scientific, Massachusetts, USA) for 30–40 min. After, cells were stained with DAPI (1:100) (4′,6-diamidino-2-phenylindole, Thermo Fisher Scientific, Massachusetts, USA) for 10 min. Finally, covers were placed on microscope slides and fluorescence images were taken using Nikon TMS microscope (Virginia, USA).

Phagocytosis determination in flow cytometry was performed in monocyte-derived DCs from donors to verify that both types of APCs efficiently capture NPs. DCs were obtained as specified in "Generation of monocyte-derived DCs" section of this article, and the doses used correspond to those previously tested in RAW 264.7.

### Cytotoxicity assay

NP cytotoxicity was assessed using the Cell Counting Kit – 8 (CCK-8) (Sigma-Aldrich, Darmstadt, Germany) by measuring viability of RAW 264.7 cells. Cells were grown in a 96-well plate and conditioned with poly(I:C) covered NPs (NP poly(I:C)) or non-covered NPs (NPs) at 1 µg/ml or 5 µg/ml poly(I:C). As a positive control, 100% viability was set to untreated cells; and as a negative control, cells cultured with 10% DMSO. After 24 h, the medium was replaced with CCK-8 solution following manufactures instructions, for at least 2 h. Then, supernatant was collected and the absorbance read at 450 nm, using 650 nm as the reference wavelength in a plate reader (Infinite^®^ 200 PRO series, Tecan Trading AG, Männedorf, Switzerland).

### Generation of monocyte-derived DCs

Heparin blood tubes were obtained from healthy donors at the University of the Basque Country (UPV/EHU). The research protocol was approved by the Ethic Committee for Research involving Human Beings (CEISH) of the UPV/EHU (M10_2022_131MR1). Volunteers gave written informed consent to use their blood samples as research material. Likewise, the handling of these cells was approved by the Research Ethics Committee with Biological Agents and Genetically Modified Organisms (CEIAB) of the UPV / EHU (M30_2022_132MR1). Peripheral blood mononuclear cells (PBMCs) were separated by Ficoll-Paque density gradient centrifugation.

Monocytes were magnetically isolated from PBMCs using anti-CD14 MicroBeads (MB), and cultured in RPMI 1640 medium with 5% AB human serum, penicillin/streptomycin (P/S), 1% L-glutamine, 200 IU/ml interleukin 4 (IL-4), and 400 IU/ml granulocyte-macrophage colony-stimulating factor (GM-CSF) (complete medium) for 5 days, at a 500,000 cell/ml and 0.5 ml/well in a 24 well plate, in order to differentiate to immature DCs (iDCs). The rest of the PBMCs were frozen and stored in liquid nitrogen with 90% serum and 10% dimethyl sulfoxide (DMSO). Medium was replaced on the 3th day with 200 IU/ml IL-4 and 800 IU/ml GM-CSF. On day 5 maturation studies were performed.

### DC maturation

On day 5, iDCs were collected and seeded in a 24 well/plate at 200,000 iDC/well, in complete medium with different maturation stimuli. The experimental conditions were: unstimulated iDC, cytokine matured DCs (mDCs), free poly(I:C), uncovered NPs and NP poly(I:C). All of them maintained 1 µg/ml or 5 µg/ml poly(I:C) in culture, and uncovered NPs were added in the same quantity as covered ones to maintain NP amount constant in all conditions. After 24h and 6h of incubation respectively, DCs were harvested. Supernatant was used for tumour necrosis factor alpha (TNF-α) enzyme-linked immunosorbent assay (ELISA) and DCs were analysed by flow cytometry (human leukocyte antigen (HLA)-DR, CD80, CD83, CD86 and CD14).

### Activation-induced marker assay

To determine an effective T cell activation against the formulations, PBMCs were cultured with NPs.

Buffy coats from healthy donors were obtained at the Basque Biobank, which were isolated from the blood of healthy donors at the Basque Transfusion Centre. The transfer of samples has been designed in accordance with the requirements of Law 14/2007, of 3 July, on Biomedical Research and Royal Decree 1716/2011, of 18 November, which establishes the basic requirements for the authorisation and operation of biobanks for biomedical research purposes and the treatment of biological samples of human origin, and regulates the operation and organisation of the National Register of Biobanks for biomedical research (CEIm CES-BIOEF 2023-06). Then, PBMCs were separated by Ficoll-Paque density gradient centrifugation and suspended at 4 × 10^6^ cell/ml in RPMI 1640 medium with 10% inactivated FBS, penicillin/streptomycin (P/S) and 1% L-glutamine. 2 × 10^6^ cells/well were cultured in a 48 well plate.

All wells were treated with 2 µg/well of anti-human CD40 (InVivoMAb, BioXcel, New Hampshire, USA) to avoid CD154 internalization. The groups studied were stimulated with 1 µM of each peptide, and for the groups without Ag, the dose of poly(I:C) or NP to be added was established based on the complete formulation. Groups used were as follows: free neoantigen peptide (Ag), free poly(I:C), nanoparticulated antigen (NP(Ag)), poly(I:C) covered NP (NP poly(I:C)) and poly(I:C) covered and antigen containing NP (NP(Ag) poly(I:C)). Both antigens, named A and B, or the NPs with antigens (NP-A and NP-B) were added simultaneously in the same groups, so for ease of understanding, they are referred to as Ag and NP(Ag), respectively. The negative control group was established with unstimulated cells, and the positive control group cells were treated with 10 µl/ml hexavalent vaccine Infanrix (EU/1/00/152/005; GlaxoSmithKline Biologicals s.a., Belgium).

The following day, supernatant was collected for interferon gamma (IFN-$\gamma$) cytokine quantification by ELISA and the surface expression markers of T cells were analysed by flow cytometry.

### Flow cytometry analysis

Maturation marker upregulation on DCs was determined by flow cytometry. First, cells were harvested and stained with the following fluorochrome-conjugated human monoclonal antibodies: anti-CD80/APC, CD83/PEVio770, CD86/FITC, CD14/PE and HLA-DR/ VioBlue (Miltenyi Biotech, Germany). Then, cells were selected with a forward vs. side scatter chart (FSC vs. SSC) gating. In addition, expression of the monocyte marker CD14 was assessed to confirm the DC phenotype. Finally, maturation markers were analysed.

In T cell analysis, PBMCs were harvested, stained with fixable far red dead cell stain kit for live/dead cell discrimination, (Thermo Fisher Scientific, USA), and further stained with the following fluorochrome-conjugated human monoclonal antibodies: anti-CD3/PEVio770, CD4/VioBlue (both from Miltenyi Biotech, Germany), CD69/FITC and CD154/PE (both from Becton Dickinson, S.A., USA). Compensation was carried out with compensation beads anti-REA (Miltenyi Biotech, Germany) and anti-Mouse Ig (Becton Dickinson, S.A., USA). A control for dead cells (heat-killed PBMCs) was included in all experiments. In analysis, singlets of living CD3^+^ cells were selected, following by a FSC vs SSC gate selection. CD3^+^ cells were then divided into CD4^+^ and CD4^−^, the latter being assumed to be CD8^+^. Activation markers for each lymphocyte type were then quantified. Activation of CD4^+^ cells was determined by CD154 and CD69 presence, and CD4^−^ cells only by CD69.

Flow cytometry was conducted on MACSQuant cytometer, and data analysis was performed using the MACSQuantify software (Miltenyi Biotech, Germany).

### ELISA from supernatants

DC and PBMC supernatants were collected, and stored at -80ºC until analysis. Cytokines were measured in each supernatant: TNF-α in DCs, and IFN-$\gamma$ for T cells. The concentration of each cytokine was measured using specific ELISA kits according to the manufacturer’s instructions (BioLegend, San Diego, CA, USA).

### Statistics

The results are expressed as mean ± standard deviation (SD) for each group. Statistical computations were performed using SPSS (IBM SPSS, Chicago, IL, USA) and GraphPad Prism 5.0 software (GraphPad Software, San Diego, CA, USA). The normal distribution of the data was checked using the Shapiro-Wilk test. For multiple comparisons, one-way ANOVA was performed, using Levene’s or Brown-Forsythe test to determine the homogeneity of variances. If homogeneous Bonferroni's Multiple Comparison post hoc test was applied, otherwise the Tamhane post-hoc test was used. For comparisons between 2 paired groups, paired Student's t-test was used when the data followed a normal distribution, while for non-normally distributed data the non-parametric Wilcoxon test was used. P-values below 0.05 were considered significant in all analyses.

## Results and discussion

### NP characterization

Biodegradable polymeric PLGA-PEI NPs were prepared by the solvent extraction-evaporation of a double emulsion (w/o/w) method. DLS was used to analyse the Z-average diameter and PdI of the NPs, and the Z-potential was determined through Laser Doppler micro-electrophoresis. Both of them measured by a Malvern^®^ Zetasizer NanoZS Model ZEN3600 (Malvern Instruments Ltd., UK). NPs exhibited homogeneous size and low PdI (below 0.2), which confirms narrow size distribution. Positive Z-potential values were also obtained, which confirms efficient PEI coating. These results are consistent with other PLGA NPs functionalised with PEI [50, 51], and meet the requirements for cellular uptake as nanometric particles have demonstrated better uptake than bigger particles [52]. Moreover, a similar encapsulation efficiency (EE%) and protein loading for both peptides were observed (Table 2), which fall within the usual range of antigen encapsulation in PLGA NPs [53, 54].

**Table 2:** Characterization of NPs

**Formulation**	**Size (nm)**	**PdI**	**Zeta potential (mV)**	**SAP (%)**	**EE (%)**	**Protein loading (%)**
NP A	249.3 ± 21.22	0.048 ± 0.048	31.2 ± 2.05	1.43 ± 0.72	53.11 ± 5.21	2.18%
NP B	245.9 ± 5.45	0.064 ± 0.024	31.9 ± 2.09	1.76 ± 0.72	56.28 ± 5.89	2.31%
NP	268.6 ± 22.2	0.144 ± 0.061	24.6 ± 2.33	-	-	-

Mean NP size (Z-average diameter), polydispersity index (PdI) and Z-potential values are determined for all NPs. For NPs containing antigens, surface absorbed protein (SAP), encapsulation efficiency (EE%) and protein loading of antigens (A and B) are also represented. Data represent mean values ± SD

### Poly(I:C) covering of NPs

**Fig. 1:**
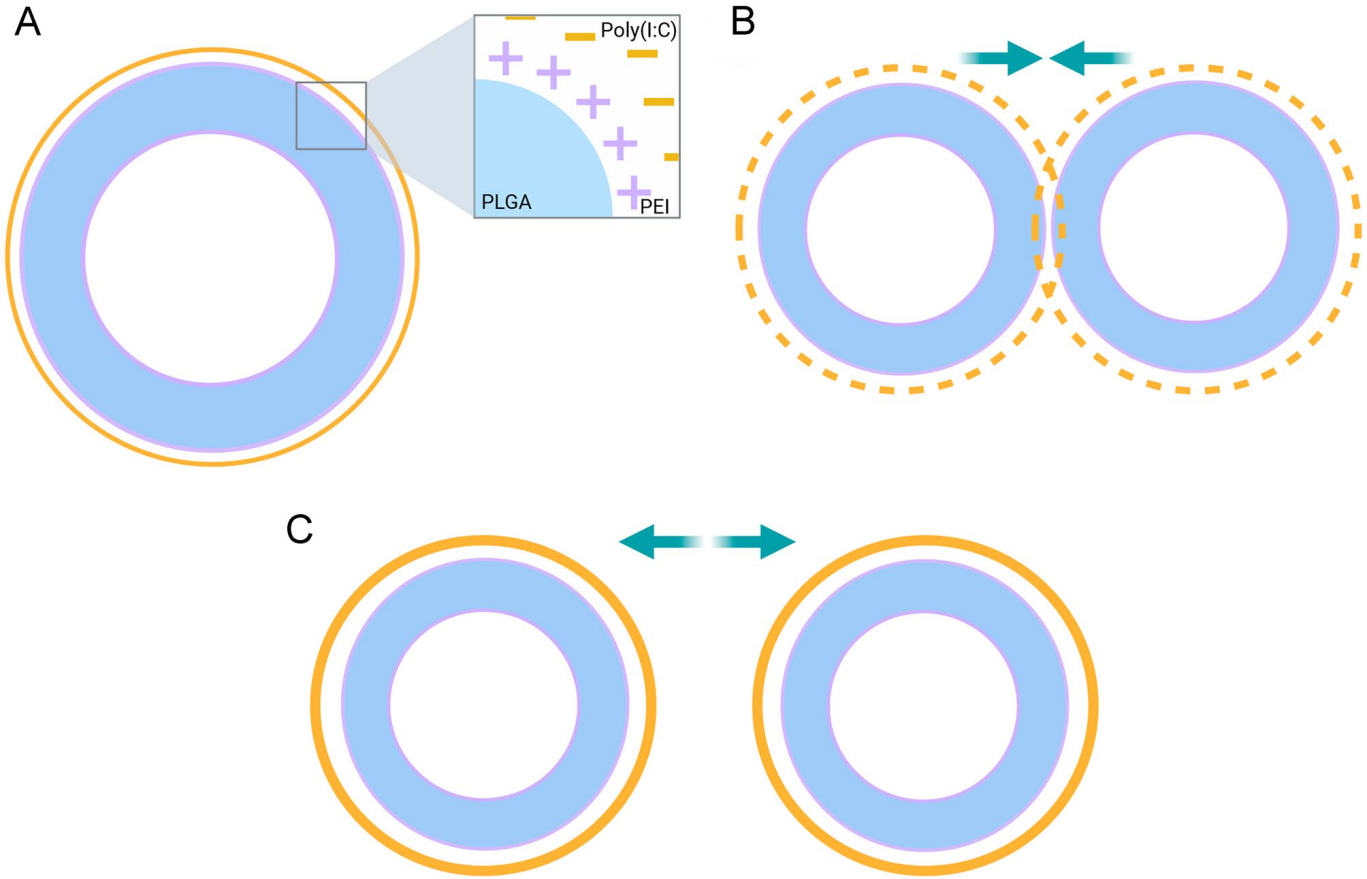
w/o/w PLGA-PEI nanoparticles with poly(I:C) covering and charge interaction diagram. **A** Positively charged PLGA-PEI NP covered with negatively charged poly(I:C). **B** Suboptimal coverage of the poly(I:C) causes the different charges of the NPs to interact with each other causing them to aggregate. **C** Complete poly(I:C) covering of the NPs. Created with BioRender.com

Poly(I:C) is a negatively charged molecule that can interact with the positively charged surface of NPs. Due to the ability of a single poly(I:C) molecule to bind with multiple NPs simultaneously, establishing the correct NP:poly(I:C) ratio is crucial to avoid NP aggregation and ensure full surface coverage (Fig. 1). To achieve this, we first determined the optimal NP concentration by using a fixed concentration of poly(I:C) and increasing concentrations of NPs. After identifying the optimal NP concentration, we then determined the best concentration of poly(I:C) by testing a range of concentrations. Size analysis was used as a means of detecting potential NP aggregation.

An important factor to bear in mind when covering the NPs is that the interaction among NPs affects the detected size. Positively charged particles covered with negatively charged poly(I:C), can interact with poly(I:C) attached to other particles. This charge interaction affects NP movement in the fluid – or Brownian motion –, and therefore, greater sizes were detected.

**Fig. 2:**
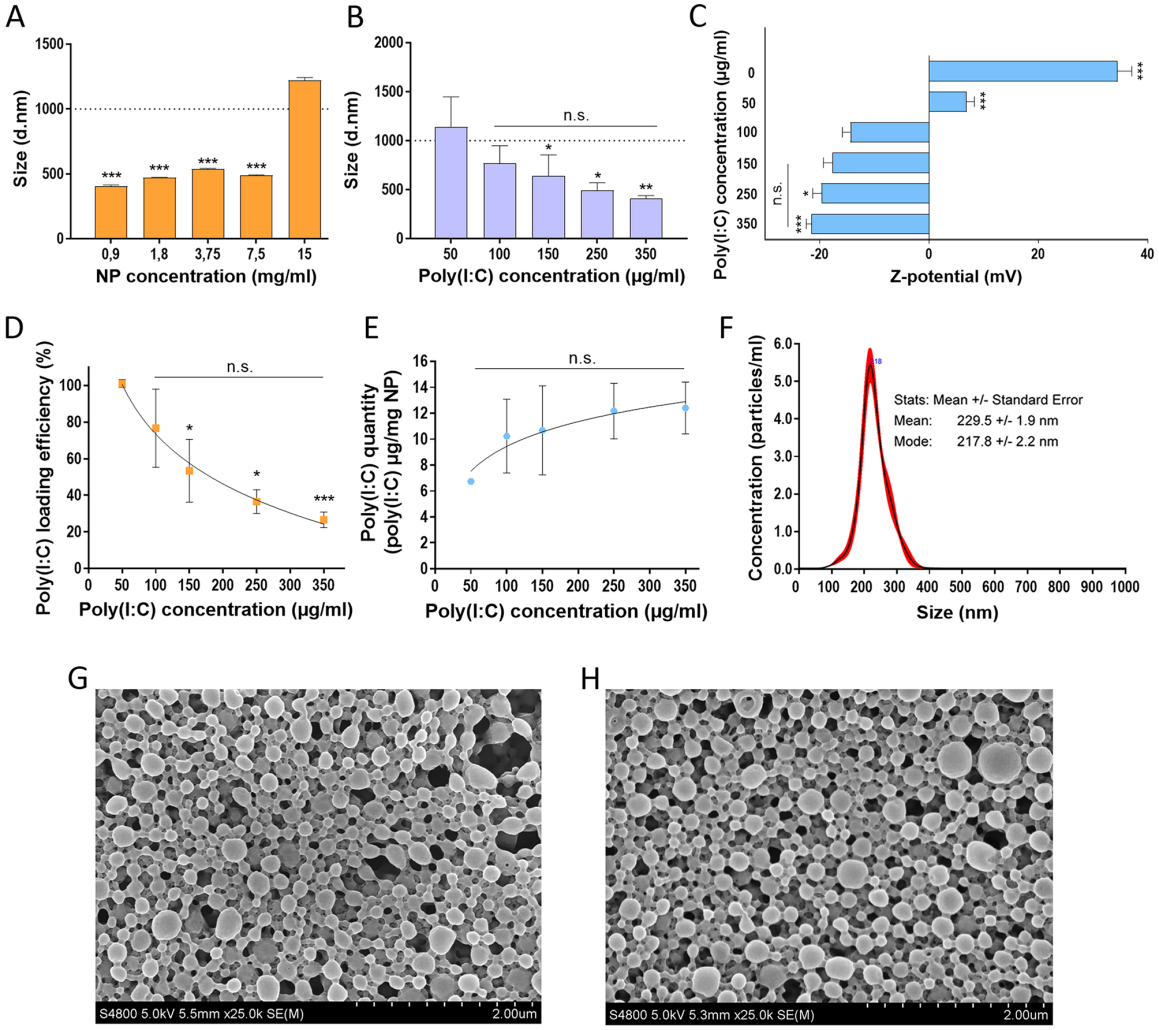
NP characterization. **A** Size of NPs with a specific concentration of poly(I:C) (150 µg/ml) (***p < 0.001 with respect to 15 mg/ml NP). **B** Size of NPs (7,5 mg/ml) coated with increasing concentrations of poly(I:C) (*p < 0.05 and *p < 0.005 with respect to 50 µg/ml poly(I:C)). **C** Z-potential of NPs (7,5 mg/ml) covered with different poly(I:C) concentrations. Increasing poly(I:C) concentrations lowered Z-potential values until no statistical differences were observed form 150 µg/ml on (*p < 0.05 and ***p < 0.001 in regard to 100 µg/ml of poly(I:C)). **D** Percentage of incorporated poly(I:C) that had been efficiently loaded onto the NPs (*p < 0.05 and ***p < 0.001 in regard to 50 µg/ml of poly(I:C)). **E** Poly(I:C) quantity loaded per 1 mg of NP. **F** Size determination of NP covered with poly(I:C) in NTA. **G, H** NP morphology with **H** or without **G** 250 µg/ml poly(I:C), in SEM

To stablish the optimal NP:poly(I:C) ratio, firstly, 150 µg/ml of poly(I:C) was used at different NP concentrations, ranging from 0.9–15 mg/ml. We observed a greater tendency for aggregation at 15 mg/ml NP concentration, which indicated high NP:poly(I:C) ratio. However, the smaller ratios significantly reduced NP aggregation, resulting in an adequate NP size (Fig. 2A). For the following experiments, the concentration of 7.5 mg/ml NPs was selected as it allowed an efficient coating of the NPs with the smallest possible volume, thus optimising the experimental conditions.

Next, poly(I:C) concentration was stablished. The NP concentration was maintained at 7.5 mg/ml and the poly(I:C) concentration ranged from 50 to 350 µg/ml. Following previous observations, low concentration was related to higher aggregation tendency due to its capacity to bind more than one NP. On the contrary, high poly(I:C) concentration allowed complete NP covering, less NP interaction, and finally, less aggregation tendency. For nano size, at least 100 µg/ml of poly(I:C) is needed (Fig. 2B). Same experiment was carried out in more acidic environments (pH 4.6) to study the best conditions for the NPs, however, we observed that an acidic pH did not allow the desired studies as it favoured the precipitation of the NPs (Supplementary Table 1). Although the pH of melanoma is slightly acidic, the intention of this vaccine is phagocytosis by APCs, with special interest in DCs, so its administration is intended to be far from the tumour to be treated, and therefore far from its acidic environment. As a result, the acidic pH was discarded for the following experiments, which is interesting as a more neutral pH would allow for subcutaneous administration.

Negatively charged poly(I:C) addition to positively charged NP decreased surface Z-potential. As in size, lower immunostimulant concentrations showed lower NP covering, leading to positive Z-potential values. While increasing poly(I:C) concentration, Z-potential decreased until around -20mV values were achieved (Fig. 2C). Results showed good covering efficiency and higher attachment in a dose dependent manner, with no statistical differences from 150 µg/ml on.

Next we wanted to determine the binding efficiency of the poly(I:C) to the NPs, as well as poly(I:C) quantity loaded per mg of NP. Concentrations below 50 µg/ml showed complete poly(I:C) attachment, while higher ones led to lower covering efficiency (Fig. 2D). Meanwhile, Fig. 2E shows an increased attachment at higher concentrations, although no statistical differences were observed. This data indicates that NP surface was completely covered with concentrations above 100 µg/ml of poly(I:C). Moreover, results were in accordance with aggregation and surface potential values (Fig. 2B, C).

Taken together, the results showed that complete covering of NPs is achieved with 7.5 mg/ml of NPs and with poly(I:C) concentrations at least of 150 µg/ml. However, more reproducible values with smaller deviation were detected with higher poly(I:C) concentrations, so following experiments were carried out with 250 µg/ml poly(I:C).

NP size alterations were confirmed with NTA (Fig. 2F) and SEM images (Fig. 2G, H), in which poly(I:C) covered NPs showed same sizes as non-covered ones. This confirms NP size was not altered in the covering process and that the size differences observed with Zetasizer equipment were due to the charge interaction between NPs.

Overall, we can conclude that poly(I:C) covering was successfully achieved by surface functionalisation of PLGA-PEI NPs.

### In vitro studies

#### NP phagocytosis and cytotoxicity studies

Phagocytosis and cytotoxicity studies were performed to ensure NPs uptake by cells and assess the potential NP toxicity.

**Fig. 3:**
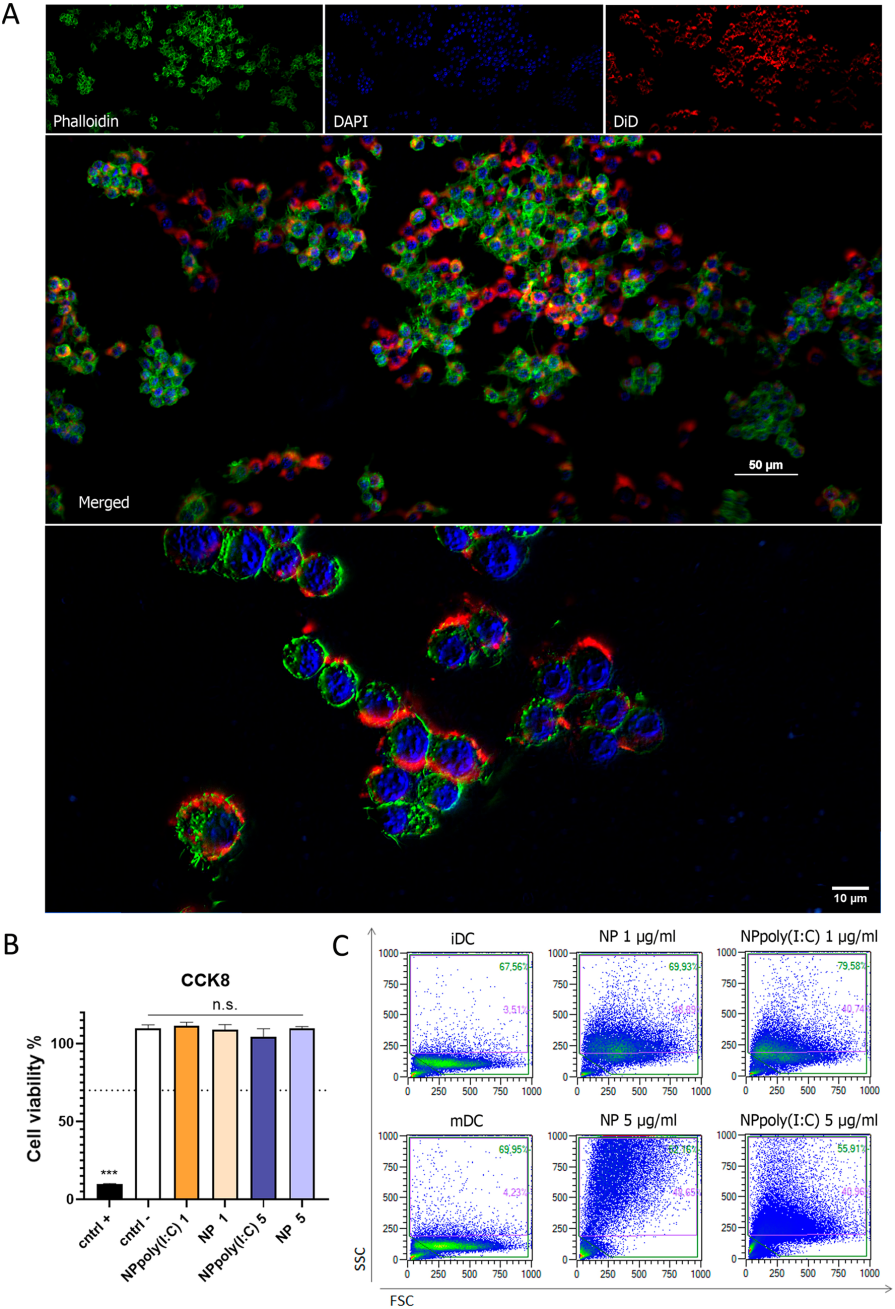
NP internalization. **A** NP internalizations observed in microscopy. RAW 264.7 cell membranes are stained in green with Alexa488-phalloidine. NPs are stained in red with DiD. Blue nucleus in DAPI. **B** Quantification of viable cells by CCK8 to determine cytotoxicity of NPs. **C** Phagocytosis of NPs by DCs was analysed by flow cytometry, in which an increase in complexation (SSC) demonstrates uptake of NPs

In phagocytosis studies, RAW 264.7 macrophage cell line was cultured with NPs dyed with DiD. After 24 h, cells were fixed in microscopy covers and stained with phalloidine and DAPI and observed under microscopy. In Fig. 3A we see the NPs in red, the cytoplasm in green and the nucleus in blue. The image shows efficient NP uptake and localization within the cytoplasm, surrounding the nucleus.

To assess NP cytocompatibility, they were incubated with RAW 264.7 cells. Cells were cultured with two different concentrations of poly(I:C) or NP poly(I:C) (1 and 5 µg/ml), and another group was included with uncoated NPs in an equivalent amount. After 24h, cell viability was measured by CCK-8 assay. The results illustrated in Fig. 3B show that none of the concentrations tested were cytotoxic (> 70%. cell viability).

Finally, cytometry analysis carried out in DCs confirmed NP uptake, in which DCs treated with NPs exhibit more complexity (SSC signal increase) (Fig. 3C). In particular, iDC and mDC groups have about 4% of the population in the complexity gating, while the NP-treated group has more than 40% of the population.

#### DC maturation

Adequate DC activation is crucial for T-cell-targeted cancer vaccines, as it allows for proper antigen presentation, and thus effective activation of the immune response [55]. Therefore, to select the most appropriate NP poly(I:C) dose, the following experiments were carried out on the target cells, i.e. dendritic cells (DCs). For this purpose, we examined whether NPs promote the up-regulation of maturation markers (HLA-DR, CD80, CD83 and CD86) and cytokine release (TNF-α) by human DCs.

Following previous experiments in RAW 264.7 cells, in which the two doses tested were non-toxic, we treated monocyte-derived iDCs with 1 and 5 µg/ml of poly(I:C), NP poly(I:C) and uncoated NP (NP). In this case, we compared the dose of 1 µg/ml poly(I:C) stimulating for 24 h, and 5 µg/ml poly(I:C) in culture for 6 h. As a maturation control, mDCs were prepared using a proinflammatory cytokine cocktail.

Results revealed that the dose of 5 µg/ml NPpoly(I:C) for 6 hours was too stimulating (Supplementary Fig. 1). A reduction in phagocytic capacity compared to uncoated cells was observed, since a decrease in SSC was apparent. In addition, the trend of the CD14 marker changed drastically because the increase in complexity was observed only in CD14^−^ cells, which were also negative for the maturation markers (Supplementary Fig. 2), and therefore, were not DCs. Furthermore, no maturation differences were detected among DCs of stimulated groups. Hence, even reducing the incubation time to 6h, a dose of NP poly(I:C) equivalent to 5 µg/ml of poly(I:C) was still too strong a stimulus for this cell type. Therefore, we concluded that 5 µg/ml NP poly(I:C) for 6 hours was not appropriate for DC stimulation.

**Fig. 4:**
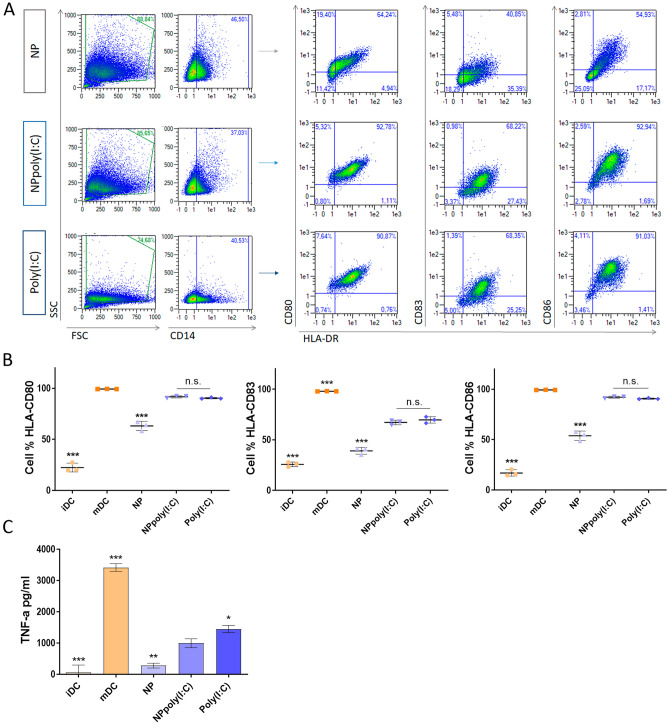
DC maturation with 1 µg/ml poly(I:C). **A** Representative flow cytometry plots from DCs after maturation with 1 µg/ml poly(I:C), NPpoly(I:C) and non-covered NPs. **B** DC maturation markers are represented in cell percentage (cell%). **C** TNF-α cytokine secretion was measured by ELISA. (*p < 0.05, **p < 0.005 and ***p < 0.001 in regard to NPpoly(I:C))

In contrast, the 1 µg/ml dose of poly(I:C) provided optimal maturation of DCs (Fig. 4A). On the one hand, the cells efficiently took up the NPs and increased their complexity accordingly. On the other hand, DCs increased maturation markers after stimulation, mainly in the poly(I:C) containing groups. In particular, compared to iDCs, non-covered NPs were able to stimulate the activation of DCs for all three markers, probably because the structure of the NPs resemble virus particles. However, with the addition of poly(I:C), the maturation capacity was enhanced. Indeed, both NPpoly(I:C) and free poly(I:C) show similar maturation to control mDCs in HLA^+^CD80^+^ and HLA^+^CD86^+^ graphs. Therefore, the addition a dose of NPpoly(I:C) equivalent to 1 µg/ml of poly(I:C) seemed to help in its purpose of maturing DCs.

Afterwards, TNF-α secretion of the 1 µg/ml poly(I:C) group was determined by ELISA. As shown in Fig. 4C, TNF-α secretion correlated with flow cytometry results since the poly(I:C)-containing groups showed the highest levels of the detected cytokine. Although the quantified amount of TNF-α in both groups were lower than the positive maturation control (mDC), the levels of TNF-α secreted appear to be sufficient to induce an adequate immune response.

Taking into account the ELISA results together with the observations made in flow cytometry, the results showed a better maturation profile with the poly(I:C)-containing groups compared to the uncovered NPs. Overall, the main conclusion is that the incorporation of poly(I:C) on NPs improves DC activation. However, it should be highlighted that although the level of DC maturation obtained with NPpoly(I:C) is similar to that achieved with free poly(I:C), it offers several advantages. On the one hand, the inclusion of the adjuvant in a nanoparticulate system manages to combine its effects and direct its immunostimulatory capacity to the same phagocytic cell, thus reducing off-target effects. On the other hand, the formulation developed allows the incorporation of another compound to direct the immune activation to the target of interest, which would allow a specific response to be achieved.

#### Specific response studies

After testing the maturation capacity of the NPs, the lymphocyte activating capacity was tested. In particular, the specific response generated by the NPs.

For this purpose, we carried out an activation-induced marker (AIM) assay in PBMCs of healthy donors: this approach allows for the identification of T cells recently activated by antigen, measured as the upregulation of CD154 and CD69 in CD4^+^ T cells; the quantification of CD69 upregulation also allows for the interrogation of the activation status of CD8^+^ T cells [56–58]. CD69 is a membrane receptor used as an early marker of activation. Its expression is low in resting lymphocytes but increases rapidly after cell activation [59]. Meanwhile, CD154 —also known as CD40L— is a transmembrane molecule that is temporarily expressed on activated CD4^+^ T cells following T-cell receptor (TCR) stimulation, making its expression antigen-dependent. CD40L interacts with CD40 expressed on antigen-presenting B cells and DCs to induce antibody formation and cellular immune responses [60].

PBMCs were incubated overnight with 1 µM of each peptide or the equivalent amount of poly(I:C) or NPs. After, cells were harvested and stained for flow cytometry analysis (Supplementary Fig. 3). Cytometry results of the activated cells were donor-dependent and varied significantly among individuals. Consequently, the results were interpreted as a ratio between the groups of interest, and the statistics were obtained by pairing the responses of each individual for both groups compared. In particular, there were three questions that needed to be addressed in this section.

**Fig. 5:**
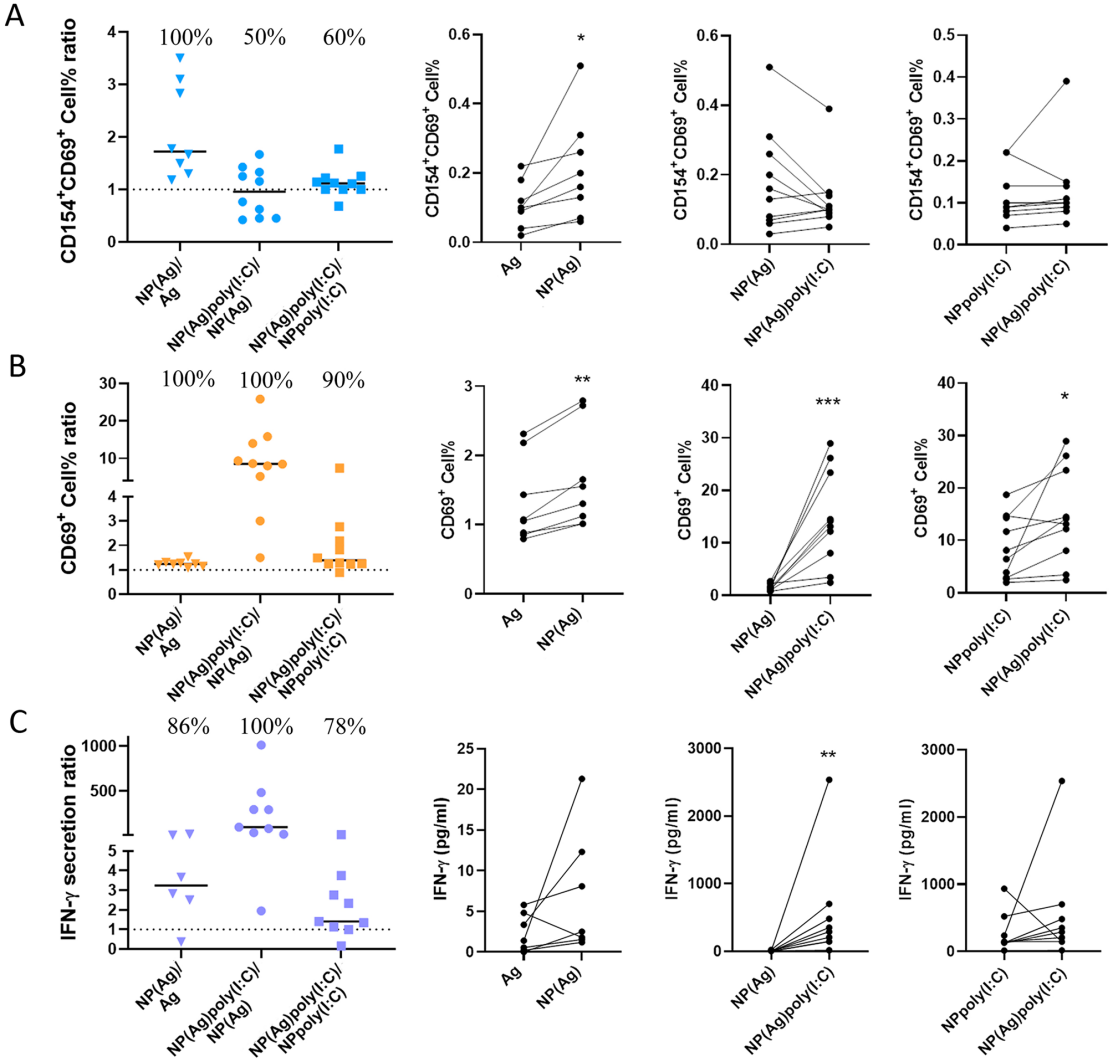
Specific response studies of T cells performed in PBMCs. Ag, NP(Ag), NPpoly(I:C) and NP(Ag)poly(I:C) effect comparison on **A** CD4^+^ T cells and **B** CD4^−^ T cells. **C** IFN-$\gamma$ cytokine release. (*p < 0.05, **p < 0.005 and ***p < 0.001)

First of all, does encapsulating the Ag enhance the lymphocyte response? The first question was assessed comparing free Ag and encapsulated Ag cytometry results (NP(Ag) vs Ag). As shown in Fig. 5A, B, after peptide encapsulation both types of T cells increased the population expressing the activation markers analysed. In particular, all donors reacted more strongly to NP(Ag) than to free Ag, probably because NPs favour antigen uptake and presentation by APCs.

Secondly, does the poly(I:C) in the formulation improve the ability to activate lymphocytes? The effect of the poly(I:C) on the activation capacity of the NPs was stablished by comparing the results of Ag containing NPs with poly(I:C) (NP(Ag)poly(I:C)) with the results of NP without poly(I:C) (NP(Ag)). As shown in Fig. 5A, B, all donors increased the percentage of activated CD4^−^ cells with NP(Ag)poly(I:C), however, there was no significant improvement on activated CD4^+^ cells. Evidence suggests that poly(I:C) acts primarily on cytotoxic lymphocytes as their activation is greatly enhanced by the addition of the adjuvant to the particle, which is of interest in cancer immunotherapy [61].

And third, is the effect enhanced by adding the Ag and the poly(I:C)? For that aim, two NPs with poly(I:C) were compared (NPpoly(I:C) vs NP(Ag)poly(I:C)), and the activation gap among them was related to the presence of the Ag in the formulation, thereby detecting the specific response. Figure 5A, B shows how the addition of the Ag to the formulation increased the percentage of activated cells, once again mainly in the CD4^−^ group with 90% of donors responding. Surprisingly, in the CD4^+^ group there was no statistical difference between the group with or without Ag in the formulation, but a modest response can be appreciated with a 60% of responding donors. However, it should be noted that the selected Ag does not come from any of the volunteers tested, so it could be expected that by using neoantigens from the patients themselves, these responses would be improved [62, 63].

Cytometry data was supplemented with an IFN-$\gamma$ ELISA. IFN-$\gamma$ is a cytokine secreted mainly by activated T-lymphocytes and its functions include: regulating the immune response, stimulating antigen presentation on MHC-I and MHC-II molecules, coordinating leukocyte-endothelium interaction, and controlling cell proliferation and apoptosis [64]. ELISA results showed that IFN-$\gamma$ secretion was highly influenced by the presence of poly(I:C). The poly(I:C) groups secreted significantly more IFN-$\gamma$ than those without adjuvant, and yet, although the presence of the Ag hints at an upward secretion trend, the groups did not differ significantly (Fig. 5C). This implies that the presence of poly(I:C) promotes the activation of lymphocytes and produces a large release of IFN-$\gamma$, and therefore favors the execution of lymphocyte functions.

In summary, nanoparticulating the neoantigen peptides and adding poly(I:C) improves the immune response by increasing the number of active cells and their degree of activation, but without affecting antigen-specific recognition, enabling personalised and effective activation of the immune system. On the other hand, the addition of poly(I:C) to the formulation enhances the lymphocyte response by CD4^−^ cells and promotes IFN-$\gamma$ secretion. This is of great importance for a complete and competent cytotoxic response [65, 66]. Compared to previously published work on poly(I:C) in PLGA particles for immunotherapy, our vaccine is the first nano-sized, PEI-functionalised, poly(I:C)-coated particles that encapsulates melanoma patient-derived neoantigens [37, 67–69]. In addition, it is the only one that tests NPs in human PBMC to analyse the specific response generated. All of this makes the developed vaccine a novelty in various aspects of particle development and experimental trial design. Nevertheless, it should be noted that the selection of the personalised neoantigen was based on the expression on the patient's tumour cells, and on the affinity with their HLA. The results obtained, however, come from PBMC from healthy volunteers, where the frequency of lymphocytes responding to these patient-derived antigens is very low. Thus, although this is a clear limitation of the assay, it is likely that in the case of patients themselves this response would be favoured [49]. In summary, the incorporation of poly(I:C) onto the peptide-based vaccine promotes and amplifies the activation of the immune response, which is expected to be greater in patients.

## Conclusion

In conclusion, NP(Ag)poly(I:C) has proven to be an interesting system for immune activation. The developed NPs were successfully coated with poly(I:C) after PEI functionalisation, and the w/o/w emulsion allowed efficient neoantigen encapsulation, which ultimately led to complete immune activation. Its effectiveness was associated to its configuration, as it produced a synergistic and combined effect mediated by its components. On the one hand, the NP acted as a vehicle for the different compounds, i.e. it offered them transport and protection, support for combining neoantigen and adjuvant, and ease of phagocytosis by APCs. On the other hand, the poly(I:C) favoured and amplified immune activation, and by being formulated in the NPs, potentiation of the immune response was achieved. Finally, the aim of the neoantigen was to direct the immune response towards the target of interest, thus enabling a personalised specific response. This demonstrates that the formulation can be a useful tool for eliciting a specific and invigorated immune response directed to tumour cells, which is the goal of interest in melanoma treatment.

## Supplementary Information

Below is the link to the electronic supplementary material.

Supplementary file1 (DOCX 934 kb)

## Data Availability

The authors are willing to provide the supporting data for the study upon a reasonable request.

## References

[1] Radiation: Ultraviolet (UV) radiation and skin cancer. World Health Organization. 2017. https://www.who.int/news-room/questions-and-answers/item/radiation-ultraviolet-(uv)-radiation-and-skin-cancer. Accessed Nov 2023.

[2] Garbe C, Amaral T, Peris K, Hauschild A, Arenberger P, Bastholt L, Bataille V, del Marmol V, Dréno B, Fargnoli MC, Grob J, Höller C, Kaufmann R, Lallas A, Lebbé C, Malvehy J, Middleton M, Moreno-Ramirez D, Pellacani G, Saiag P, Stratigos AJ, Vieira R, Zalaudek I, Eggermont AMM. European consensus-based interdisciplinary guideline for melanoma. Part 1: Diagnostics – Update 2019. Eur J Cancer. 2020;126:141–58. 10.1016/j.ejca.2019.11.014. 31928887 10.1016/j.ejca.2019.11.014

[3] Garbe C, Peris K, Hauschild A, Saiag P, Middleton M, Bastholt L, Grob J, Malvehy J, Newton-Bishop J, Stratigos AJ, Pehamberger H, Eggermont AM. Diagnosis and treatment of melanoma. European consensus-based interdisciplinary guideline - Update 2016. Eur J Cancer. 2016;63:201–17. 10.1016/j.ejca.2016.05.005. 27367293 10.1016/j.ejca.2016.05.005

[4] Albittar AA, Alhalabi O, Glitza Oliva IC. Immunotherapy for Melanoma. Adv Exp Med Biol. 2020;1244:51–68. 10.1007/978-3-030-41008-7_3. 32301010 10.1007/978-3-030-41008-7_3

[5] Martins F, Sofiya L, Sykiotis GP, Lamine F, Maillard M, Fraga M, Shabafrouz K, Ribi C, Cairoli A, Guex-Crosier Y, Kuntzer T, Michielin O, Peters S, Coukos G, Spertini F, Thompson JA, Obeid M. Adverse effects of immune-checkpoint inhibitors: Epidemiology, management and surveillance. Nat Rev Clin Oncol. 2019;16:563–80. 10.1038/s41571-019-0218-0. 31092901 10.1038/s41571-019-0218-0

[6] Morgado M, Plácido A, Morgado S, Roque F. Management of the adverse effects of immune checkpoint inhibitors. Vaccines (Basel). 2020;8:575. 10.3390/vaccines8040575. 33019641 10.3390/vaccines8040575 PMC7711557

[7] Schoenfeld AJ, Hellmann MD. Acquired resistance to immune checkpoint inhibitors. Cancer Cell. 2020;37:443–55. 10.1016/j.ccell.2020.03.017. 32289269 10.1016/j.ccell.2020.03.017 PMC7182070

[8] Wróbel S, Przybyło M, Stępień E. The clinical trial landscape for melanoma therapies. J Clin Med. 2019;8:368. 10.3390/jcm8030368. 30884760 10.3390/jcm8030368 PMC6463026

[9] Hirayama M, Nishimura Y. The present status and future prospects of peptide-based cancer vaccines. Int Immunol. 2016;28:319–28. 10.1093/intimm/dxw027. 27235694 10.1093/intimm/dxw027

[10] Types of biological therapy. In: SEER training. National Cancer Institute. https://training.seer.cancer.gov/treatment/biotherapy/immunotherapy.html. Accessed Nov 2023.

[11] Wu Z, Man S, Sun R, Li Z, Wu Y, Zuo D. Recent advances and challenges of immune checkpoint inhibitors in immunotherapy of non-small cell lung cancer. Int Immunopharmacol. 2020;85:106613. 10.1016/j.intimp.2020.106613. 32450531 10.1016/j.intimp.2020.106613

[12] Chen DS, Mellman I. Elements of cancer immunity and the cancer-immune set point. Nature. 2017;541:321–30. 10.1038/nature21349. 28102259 10.1038/nature21349

[13] Maeng HM, Berzofsky JA. Strategies for developing and optimizing cancer vaccines. F1000Res. 2019;8(F1000 Faculty Rev):654. 10.12688/f1000research.18693.1. 10.12688/f1000research.18693.1 PMC6518434 31131086

[14] Sahin U, Türeci Ö. Personalized vaccines for cancer immunotherapy. Science. 2018;359:1355–60. 10.1126/science.aar7112. 29567706 10.1126/science.aar7112

[15] Bezu L, Kepp O, Cerrato G, Pol J, Fucikova J, Spisek R, Zitvogel L, Kroemer G, Galluzzi L. Trial watch: Peptide-based vaccines in anticancer therapy. Oncoimmunology. 2018;7:e1511506. 10.1080/2162402X.2018.1511506. 30524907 10.1080/2162402X.2018.1511506 PMC6279318

[16] Lang F, Schrörs B, Löwer M, Türeci Ö, Sahin U. Identification of neoantigens for individualised cancer immunotherapy. Nat Rev Drug Discovery. 2022;21:261–82. 10.1038/s41573-021-00387-y. 35105974 10.1038/s41573-021-00387-y PMC7612664

[17] Türeci Ö, Vormehr M, Diken M, Kreiter S, Huber C, Sahin U. Targeting the heterogeneity of cancer with individualized neoepitope vaccines. Clin Cancer Res. 2016;22:1885–96. 10.1158/1078-0432.CCR-15-1509. 27084742 10.1158/1078-0432.CCR-15-1509

[18] Hu Z, Leet DE, Allesøe RL, Oliveira G, Li S, Luoma AM, Liu J, Forman J, Huang T, Iorgulescu JB, Holden R, Sarkizova S, Gohil SH, Redd RA, Sun J, Elagina L, Giobbie-Hurder A, Zhang W, Peter L, Ciantra Z, Rodig S, Olive O, Shetty K, Pyrdol J, Uduman M, Lee PC, Bachireddy P, Buchbinder EI, Yoon CH, Neuberg D, Pentelute BL, Hacohen N, Livak KJ, Shukla SA, Olsen LR, Barouch DH, Wucherpfennig KW, Fritsch EF, Keskin DB, Wu CJ, Ott PA. Personal neoantigen vaccines induce persistent memory T cell responses and epitope spreading in patients with melanoma. Nat Med. 2021;27:515–25. 10.1038/s41591-020-01206-4. 33479501 10.1038/s41591-020-01206-4 PMC8273876

[19] Schneble E, Clifton GT, Hale DF, Peoples GE. Peptide-based cancer vaccine strategies and clinical results. In: Anonymous methods in molecular biology (Clifton, N.J.). New York, NY: Springer New York; 2016. p. 797–817. 10.1007/978-1-4939-3387-7_46 27076168

[20] Coulie PG, Van den Eynde BJ, van der Bruggen P, Boon T. Tumour antigens recognized by T lymphocytes: At the core of cancer immunotherapy. Nature reviews. Cancer. 2014;14:135–46. 10.1038/nrc3670. 24457417 10.1038/nrc3670

[21] Ma M, Liu J, Jin S, Wang L. Development of tumour peptide vaccines: From universalization to personalization. Scand J Immunol. 2020;91:e12875. 10.1111/sji.12875. 32090366 10.1111/sji.12875

[22] Liu J, Fu M, Wang M, Wan D, Wei Y, Wei X. Cancer vaccines as promising immuno-therapeutics: Platforms and current progress. J Hematol Oncol. 2022;15:28. 10.1186/s13045-022-01247-x. 35303904 10.1186/s13045-022-01247-x PMC8931585

[23] Fosgerau K, Hoffmann T. Peptide therapeutics: current status and future directions. Drug Discov Today. 2015;20:122–8. 10.1016/j.drudis.2014.10.003. 25450771 10.1016/j.drudis.2014.10.003

[24] Pennock ND, White JT, Cross EW, Cheney EE, Tamburini BA, Kedl RM. T cell responses: Naïve to memory and everything in between. Adv Physiol Educ. 2013;37:273–83. 10.1152/advan.00066.2013. 24292902 10.1152/advan.00066.2013 PMC4089090

[25] Khong H, Overwijk WW. Adjuvants for peptide-based cancer vaccines. J Immunother Cancer. 2016;4:56. 10.1186/s40425-016-0160-y. 27660710 10.1186/s40425-016-0160-y PMC5028954

[26] Schlosser E, Mueller M, Fischer S, Basta S, Busch DH, Gander B, Groettrup M. TLR ligands and antigen need to be coencapsulated into the same biodegradable microsphere for the generation of potent cytotoxic T lymphocyte responses. Vaccine. 2008;26:1626–37. 10.1016/j.vaccine.2008.01.030. 18295941 10.1016/j.vaccine.2008.01.030

[27] Mutwiri G, Gerdts V, van Drunen Littel-van den Hurk S, Auray G, Eng N, Garlapati S, Babiuk LA, Potter A. Combination adjuvants: The next generation of adjuvants? Expert Rev Vaccines. 2011;10:95–107. 10.1586/erv.10.154. 21162624 10.1586/erv.10.154

[28] Moon JJ, Huang B, Irvine DJ. Engineering nano- and microparticles to tune immunity. Adv Mater (Weinheim). 2012;24:3724–46. 10.1002/adma.201200446. 10.1002/adma.201200446 PMC3786137 22641380

[29] De Temmerman M, Rejman J, Demeester J, Irvine DJ, Gander B, De Smedt SC. Particulate vaccines: On the quest for optimal delivery and immune response. Drug Discov Today. 2011;16:569–82. 10.1016/j.drudis.2011.04.006. 21570475 10.1016/j.drudis.2011.04.006

[30] Pal I, Ramsey JD. The role of the lymphatic system in vaccine trafficking and immune response. Adv Drug Deliv Rev. 2011;63:909–22. 10.1016/j.addr.2011.05.018. 21683103 10.1016/j.addr.2011.05.018

[31] Royal RE, Vence LM, Wray T, Cormier JN, Lee JE, Gershenwald JE, Ross MI, Wargo JA, Amaria RN, Davies MA, Diab A, Glitza IC, Hwu W, Patel SP, Woodman SE, Overwijk WW, Hwu P. A toll-like receptor agonist to drive melanoma regression as a vaccination adjuvant or by direct tumor application. J Clin Oncol. 2017;35:9582. 10.1200/JCO.2017.35.15_suppl.9582.

[32] Mbow ML, De Gregorio E, Valiante NM, Rappuoli R. New adjuvants for human vaccines. Curr Opin Immunol. 2010;22:411–6. 10.1016/j.coi.2010.04.004. 20466528 10.1016/j.coi.2010.04.004

[33] Vasir JK, Labhasetwar V. Biodegradable nanoparticles for cytosolic delivery of therapeutics. Adv Drug Deliv Rev. 2007;59:718–28. 10.1016/j.addr.2007.06.003. 17683826 10.1016/j.addr.2007.06.003 PMC2002520

[34] Su Y, Zhang B, Sun R, Liu W, Zhu Q, Zhang X, Wang R, Chen C. PLGA-based biodegradable microspheres in drug delivery: recent advances in research and application. Drug Deliv. 2021;28:1397–418. 10.1080/10717544.2021.1938756. 34184949 10.1080/10717544.2021.1938756 PMC8248937

[35] Allahyari M, Mohit E. Peptide/protein vaccine delivery system based on PLGA particles. Hum Vaccin Immunother. 2016;12:806–28. 10.1080/21645515.2015.1102804. 26513024 10.1080/21645515.2015.1102804 PMC4964737

[36] Lü J, Liang Z, Wang X, Gu J, Yao Q, Chen C. New polymer of lactic-co-glycolic acid-modified polyethylenimine for nucleic acid delivery. Nanomedicine (Lond). 2016;11:1971–91. 10.2217/nnm-2016-0128. 27456396 10.2217/nnm-2016-0128 PMC4996154

[37] Salvador A, Sandgren KJ, Liang F, Thompson EA, Koup RA, Pedraz JL, Hernandez RM, Loré K, Igartua M. Design and evaluation of surface and adjuvant modified PLGA microspheres for uptake by dendritic cells to improve vaccine responses. Int J Pharm. 2015;496:371–81. 10.1016/j.ijpharm.2015.10.037. 26475970 10.1016/j.ijpharm.2015.10.037

[38] Mahjub R, Jatana S, Lee SE, Qin Z, Pauli G, Soleimani M, Madadi S, Li S. Recent advances in applying nanotechnologies for cancer immunotherapy. J Control Release. 2018;288:239–63. 10.1016/j.jconrel.2018.09.010. 30223043 10.1016/j.jconrel.2018.09.010

[39] Yang Z, Ma Y, Zhao H, Yuan Y, Kim BYS. Nanotechnology platforms for cancer immunotherapy. Wiley Interdiscip Rev Nanomed Nanobiotechnol. 2020;12:e1590. 10.1002/wnan.1590. 31696664 10.1002/wnan.1590

[40] Horvath D, Basler M. PLGA particles in immunotherapy. Pharmaceutics. 2023;15:615. 10.3390/pharmaceutics15020615. 36839937 10.3390/pharmaceutics15020615 PMC9965784

[41] Hafner AM, Corthésy B, Merkle HP. Particulate formulations for the delivery of poly(I:C) as vaccine adjuvant. Adv Drug Deliv Rev. 2013;65:1386–99. 10.1016/j.addr.2013.05.013. 23751781 10.1016/j.addr.2013.05.013

[42] Longhi MP, Trumpfheller C, Idoyaga J, Caskey M, Matos I, Kluger C, Salazar AM, Colonna M, Steinman RM. Dendritic cells require a systemic type I interferon response to mature and induce CD4+ Th1 immunity with poly IC as adjuvant. J Exp Med. 2009;206:1589–602. 10.1084/jem.20090247. 19564349 10.1084/jem.20090247 PMC2715098

[43] Verdijk RM, Mutis T, Esendam B, Kamp J, Melief CJ, Brand A, Goulmy E. Polyriboinosinic polyribocytidylic acid (Poly(I:C)) induces stable maturation of functionally active human dendritic cells. J Immunol. 1950;1999(163):57–61. 10.4049/jimmunol.163.1.57. 10384099

[44] Cella M, Salio M, Sakakibara Y, Langen H, Julkunen I, Lanzavecchia A. Maturation, activation, and protection of dendritic cells induced by double-stranded RNA. J Exp Med. 1999;189:821–9. 10.1084/jem.189.5.821. 10049946 10.1084/jem.189.5.821 PMC2192946

[45] Salem ML, Kadima AN, Cole DJ, Gillanders WE. Defining the antigen-specific T-cell response to vaccination and poly(I:C) /tlr3 signaling: Evidence of enhanced primary and memory CD8 T-cell responses and antitumor immunity. J Immunother. 1997;2005(28):220–8. 10.1097/01.cji.0000156828.75196.0d. 10.1097/01.cji.0000156828.75196.0d 15838378

[46] De Waele J, Verhezen T, van der Heijden S, Berneman ZN, Peeters M, Lardon F, Wouters A, Smits ELJM. A systematic review on poly(I:C) and poly-ICLC in glioblastoma: Adjuvants coordinating the unlocking of immunotherapy. J Exp Clin Cancer Res. 2021;40:1–213. 10.1186/s13046-021-02017-2. 34172082 10.1186/s13046-021-02017-2 PMC8229304

[47] Chen S, Lv M, Fang S, Ye W, Gao Y, Xu Y. Poly(I:C) enhanced anti-cervical cancer immunities induced by dendritic cells-derived exosomes. Int J Biol Macromol. 2018;113:1182–7. 10.1016/j.ijbiomac.2018.02.034. 29427678 10.1016/j.ijbiomac.2018.02.034

[48] Di S, Zhou M, Pan Z, Sun R, Chen M, Jiang H, Shi B, Luo H, Li Z. Combined adjuvant of poly I: C improves antitumor effects of CAR-T cells. Front Oncol. 2019;9:241. 10.3389/fonc.2019.00241. 31058074 10.3389/fonc.2019.00241 PMC6481273

[49] Malaina I, Gonzalez-Melero L, Martínez L, Salvador A, Sanchez-Diez A, Asumendi A, Margareto J, Carrasco-Pujante J, Legarreta L, García MA, Pérez-Pinilla MB, Izu R, Martínez de la Fuente I, Igartua M, Alonso S, Hernandez RM, Boyano MD. Computational and experimental evaluation of the immune response of neoantigens for personalized vaccine design. Int J Mol Sci. 2023;24:9024. 10.3390/ijms24109024. 37240369 10.3390/ijms24109024 PMC10219310

[50] Bivas-Benita M, Romeijn S, Junginger HE, Borchard G. PLGA-PEI nanoparticles for gene delivery to pulmonary epithelium. Eur J Pharm Biopharm. 2004;58:1–6. 10.1016/j.ejpb.2004.03.008. 15207531 10.1016/j.ejpb.2004.03.008 PMC7127346

[51] Gu P, Wusiman A, Wang S, Zhang Y, Liu Z, Hu Y, Liu J, Wang D. Polyethylenimine-coated PLGA nanoparticles-encapsulated Angelica sinensis polysaccharide as an adjuvant to enhance immune responses. Carbohyd Polym. 2019;223:115128. 10.1016/j.carbpol.2019.115128. 10.1016/j.carbpol.2019.115128 31427012

[52] Panyam J, Labhasetwar V. Biodegradable nanoparticles for drug and gene delivery to cells and tissue. Adv Drug Deliv Rev. 2003;55:329–47. 10.1016/S0169-409X(02)00228-4. 12628320 10.1016/s0169-409x(02)00228-4

[53] Zhang Z, Tongchusak S, Mizukami Y, Kang YJ, Ioji T, Touma M, Reinhold B, Keskin DB, Reinherz EL, Sasada T. Induction of anti-tumor cytotoxic T cell responses through PLGA-nanoparticle mediated antigen delivery. Biomaterials. 2011;32:3666–78. 10.1016/j.biomaterials.2011.01.067. 21345488 10.1016/j.biomaterials.2011.01.067

[54] Iranpour S, Nejati V, Delirezh N, Biparva P, Shirian S. Enhanced stimulation of anti-breast cancer T cells responses by dendritic cells loaded with poly lactic-co-glycolic acid (PLGA) nanoparticle encapsulated tumor antigens. J Exp Clin Cancer Res. 2016;35:168. 10.1186/s13046-016-0444-6. 27782834 10.1186/s13046-016-0444-6 PMC5080692

[55] Banchereau J, Steinman RM. Dendritic cells and the control of immunity. Nature (London). 1998;392:245–52. 10.1038/32588. 9521319 10.1038/32588

[56] Frentsch M, Arbach O, Kirchhoff D, Moewes B, Worm M, Rothe M, Scheffold A, Thiel A. Direct access to CD4+ T cells specific for defined antigens according to CD154 expression. Nat Med. 2005;11:1118–24. 10.1038/nm1292. 16186818 10.1038/nm1292

[57] Chattopadhyay P, Yu J, Roederer M. A live-cell assay to detect antigen-specific CD4+ T cells with diverse cytokine profiles. Nat Med. 2005;11:1113–7. 10.1038/nm1293. 16186817 10.1038/nm1293

[58] Gomez-Tourino I, Kamra Y, Baptista R, Lorenc A, Peakman M. T cell receptor β-chains display abnormal shortening and repertoire sharing in type 1 diabetes. Nat Commun. 2017;8:1792–815. 10.1038/s41467-017-01925-2. 29176645 10.1038/s41467-017-01925-2 PMC5702608

[59] González-Amaro R, Cortés JR, Sánchez-Madrid F, Martín P. Is CD69 an effective brake to control inflammatory diseases? Trends Mol Med. 2013;19:625–32. 10.1016/j.molmed.2013.07.006. 23954168 10.1016/j.molmed.2013.07.006 PMC4171681

[60] Jiang H, Chess L. An integrated view of suppressor T cell subsets in immunoregulation. J Clin Investig. 2004;114:1198–208. 10.1172/JCI23411. 15520848 10.1172/JCI23411 PMC524238

[61] Raskov H, Orhan A, Christensen JP, Gögenur I. Cytotoxic CD8+ T cells in cancer and cancer immunotherapy. Br J Cancer. 2021;124:359–67. 10.1038/s41416-020-01048-4. 32929195 10.1038/s41416-020-01048-4 PMC7853123

[62] Nelde A, Walz JS, Kowalewski DJ, Schuster H, Wolz O, Peper JK, Cardona Gloria Y, Langerak AW, Muggen AF, Claus R, Bonzheim I, Fend F, Salih HR, Kanz L, Rammensee H, Stevanović S, Weber ANR. HLA class I-restricted MYD88 L265P-derived peptides as specific targets for lymphoma immunotherapy. Oncoimmunology. 2017;6:e1219825. 10.1080/2162402X.2016.1219825. 28405493 10.1080/2162402X.2016.1219825 PMC5384368

[63] Ott PA, Hu Z, Keskin DB, Shukla SA, Sun J, Bozym DJ, Zhang W, Luoma A, Giobbie-Hurder A, Peter L, Chen C, Olive O, Carter TA, Li S, Lieb DJ, Eisenhaure T, Gjini E, Stevens J, Lane WJ, Javeri I, Nellaiappan K, Salazar AM, Daley H, Seaman M, Buchbinder EI, Yoon CH, Harden M, Lennon N, Gabriel S, Rodig SJ, Barouch DH, Aster JC, Getz G, Wucherpfennig K, Neuberg D, Ritz J, Lander ES, Fritsch EF, Hacohen N, Wu CJ. An immunogenic personal neoantigen vaccine for patients with melanoma. Nature (London). 2017;547:217–21. 10.1038/nature22991. 28678778 10.1038/nature22991 PMC5577644

[64] Boehm U, Klamp T, Groot M, Howard JC. Cellular responses to interferon-γ. Annu Rev Immunol. 1997;15:749–95. 10.1146/annurev.immunol.15.1.749. 9143706 10.1146/annurev.immunol.15.1.749

[65] Knutson KL, Disis ML. Tumor antigen-specific T helper cells in cancer immunity and immunotherapy. Cancer Immunol Immunother. 2005;54:721–8. 10.1007/s00262-004-0653-2. 16010587 10.1007/s00262-004-0653-2 PMC11032889

[66] Haabeth OAW, Lorvik KB, Hammarström C, Donaldson IM, Haraldsen G, Bogen B, Corthay A. Inflammation driven by tumour-specific Th1 cells protects against B-cell cancer. Nat Commun. 2011;2:240. 10.1038/ncomms1239. 21407206 10.1038/ncomms1239 PMC3072106

[67] Hafner AM, Corthésy B, Textor M, Merkle HP. Surface-assembled poly(I:C) on PEGylated PLGA microspheres as vaccine adjuvant: APC activation and bystander cell stimulation. Int J Pharm. 2016;514:176–88. 10.1016/j.ijpharm.2016.07.042. 27863662 10.1016/j.ijpharm.2016.07.042

[68] Wischke C, Zimmermann J, Wessinger B, Schendler A, Borchert HH, Peters JH, Nesselhut T, Lorenzen DR. Poly(I:C) coated PLGA microparticles induce dendritic cell maturation. Int J Pharm. 2009;365:61–8. 10.1016/j.ijpharm.2008.08.039. 18812217 10.1016/j.ijpharm.2008.08.039

[69] Du X, Tan D, Gong Y, Zhang Y, Han J, Lv W, Xie T, He P, Hou Z, Xu K, Tan J, Zhu B. A new poly(I:C)-decorated PLGA-PEG nanoparticle promotes Mycobacterium tuberculosis fusion protein to induce comprehensive immune responses in mice intranasally. Microb Pathog. 2022;162:105335. 10.1016/j.micpath.2021.105335. 34861347 10.1016/j.micpath.2021.105335

