# Supplementary material for: PLGA-PEI nanoparticle covered with poly(I:C) for personalised cancer immunotherapy
Source: Drug Deliv Transl Res. 2024 Mar 1;14(10):2788–803. doi: 10.1007/s13346-024-01557-2 (PMC11525302; doi:10.1007/s13346-024-01557-2)
Supplement: Supplementary file 1 — Supplementary file1 (DOCX 934 kb) [file 13346_2024_1557_MOESM1_ESM.docx]

**PLGA-PEI nanoparticle covered with poly(I:C) for personalised cancer immunotherapy**

**Drug Delivery and Translational Research (DDTR)**

Lorena Gonzalez-Melero^1,2†^, Edorta Santos-Vizcaino^1,2,3†^, Rubén Varela-Calvino^4^, Iria Gomez-Tourino^5,6^, Aintzane Asumendi^7,8^, Maria Dolores Boyano^7,8^, Manoli Igartua^1,2,3^*, Rosa Maria Hernandez^1,2,3^*

^1^NanoBioCel Research Group, Laboratory of Pharmaceutics, School of Pharmacy, University of the Basque Country (UPV/EHU), Vitoria-Gasteiz, Spain.

^2^Bioaraba, NanoBioCel Research Group, Vitoria-Gasteiz, Spain.

^3^Biomedical Research Networking Centre in Bioengineering, Biomaterials and Nanomedicine (CIBER-BBN). Institute of Health Carlos III, Madrid, Spain.

^4^Department of Biochemistry and Molecular Biology, School of Pharmacy, University of Santiago de Compostela, Santiago, Spain.

^5^Centre for Research in Molecular Medicine and Chronic Diseases (CiMUS), University of Santiago de Compostela, Santiago, Spain.

^6^Health Research Institute of Santiago de Compostela (IDIS), Santiago, Spain.

^7^Biocruces Bizkaia Health Research Institute, 48903 Barakaldo, Spain.

^8^Department of Cell Biology and Histology, Faculty of Medicine and Nursing, University of the Basque Country (UPV/EHU), 48940 Leioa, Spain.

*co-correspondings: rosa.hernandez@ehu.eus; manoli.igartua@ehu.eus

^†^These two authors contributed equally to this work.

**Supplementary Table 1.** Mean NP size (Z-average diameter), polydispersity index (PDI) and Z-potential values for NPs covered at pH 4.6 with 0-350 µg/ml poly(I:C) shows high NP sizes and suboptimal PdI values.

| **Formulation** | **Size (nm)** | **PdI** | **Zeta potential (mV)** |
| --- | --- | --- | --- |
| NP PLGA PEI - pH 4,6 – 0 µg/ml poly(I:C) | 328,9 ± 1,4 | 0,309 ± 0,018 | 8,37 ± 0,17 |
| NP PLGA PEI - pH 4,6 - 150 µg/ml poly(I:C) | 2347,0 ± 388,38 | 0,855 ± 0,108 | -8,59 ± 0,04 |
| NP PLGA PEI - pH 4,6 - 250 µg/ml poly(I:C) | 772,3 ± 52,1 | 0,522 ± 0,066 | -19,70 ± 0,87 |
| NP PLGA PEI - pH 4,6 - 350 µg/ml poly(I:C) | 608,6 ± 47,69 | 0,407 ± 0,003 | -26,71 ± 0,36 |

**
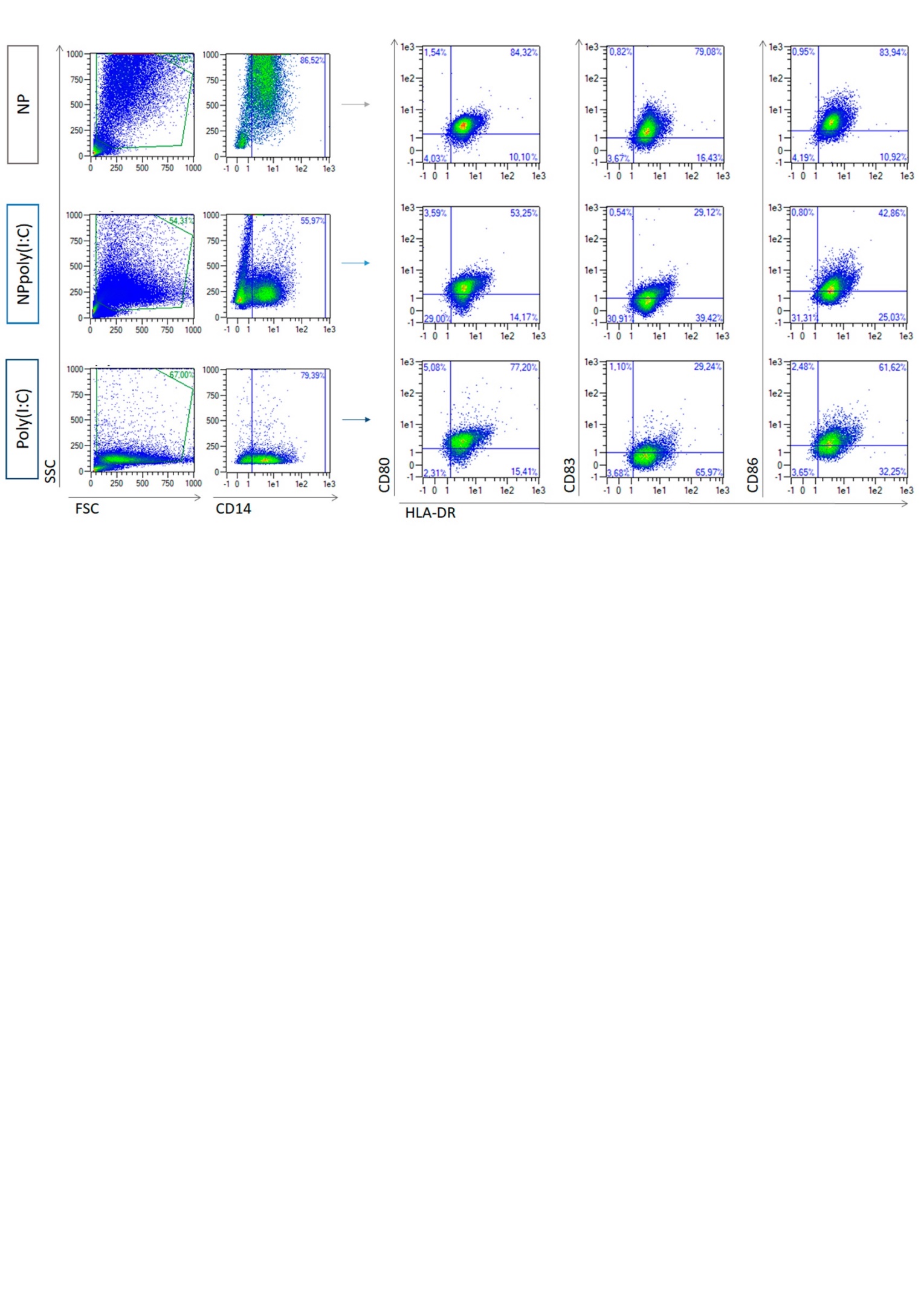
**

**Supplementary figure 1.** Representative flow cytometry plots from DCs after maturation with 5 µg/ml poly(I:C), NPpoly(I:C) and non-covered NPs.


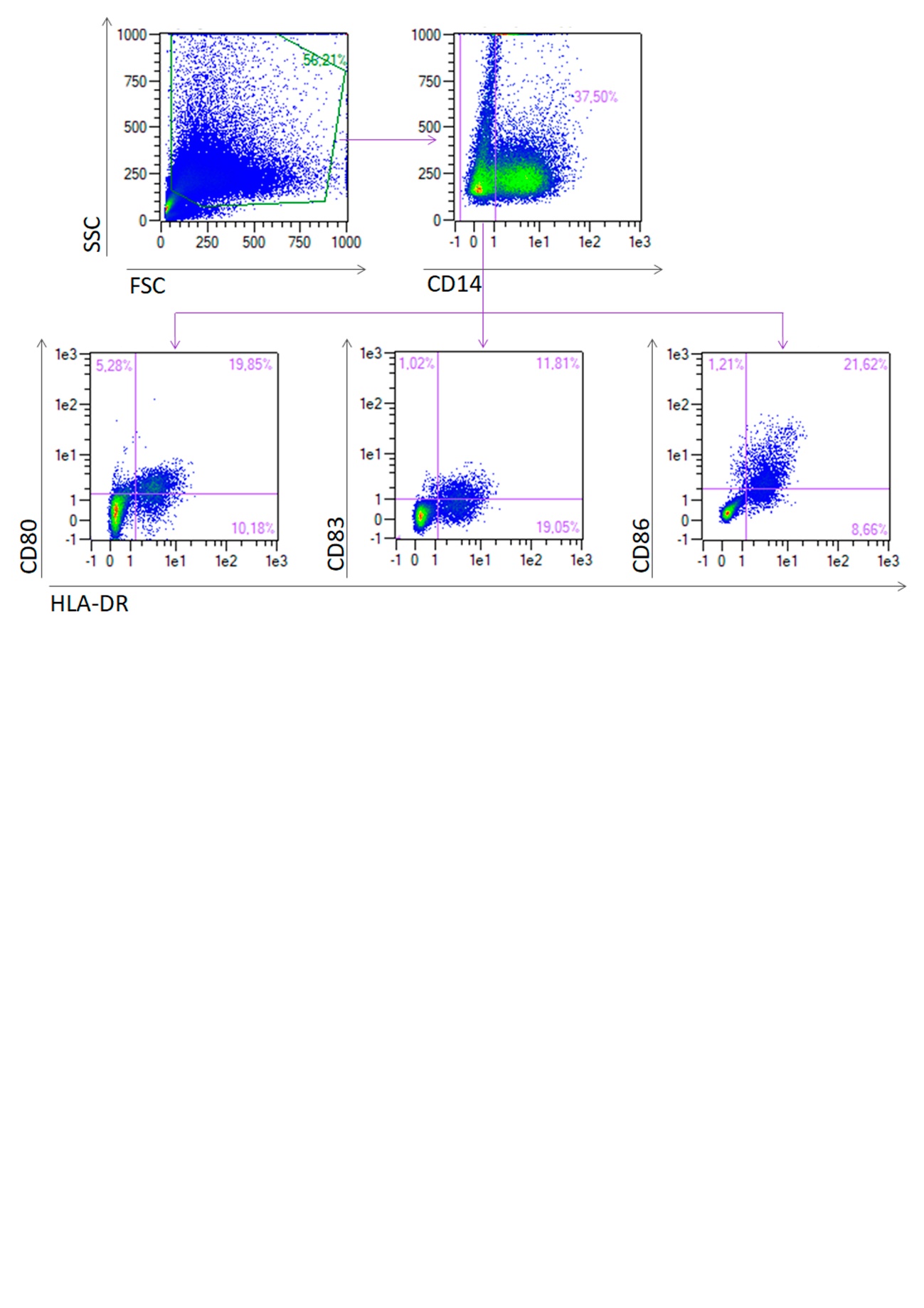
**Supplementary figure 2.** Representative flow cytometry plots from CD14^-^ cells after maturation with NPpoly(I:C) at 5 µg/ml poly(I:C).

**
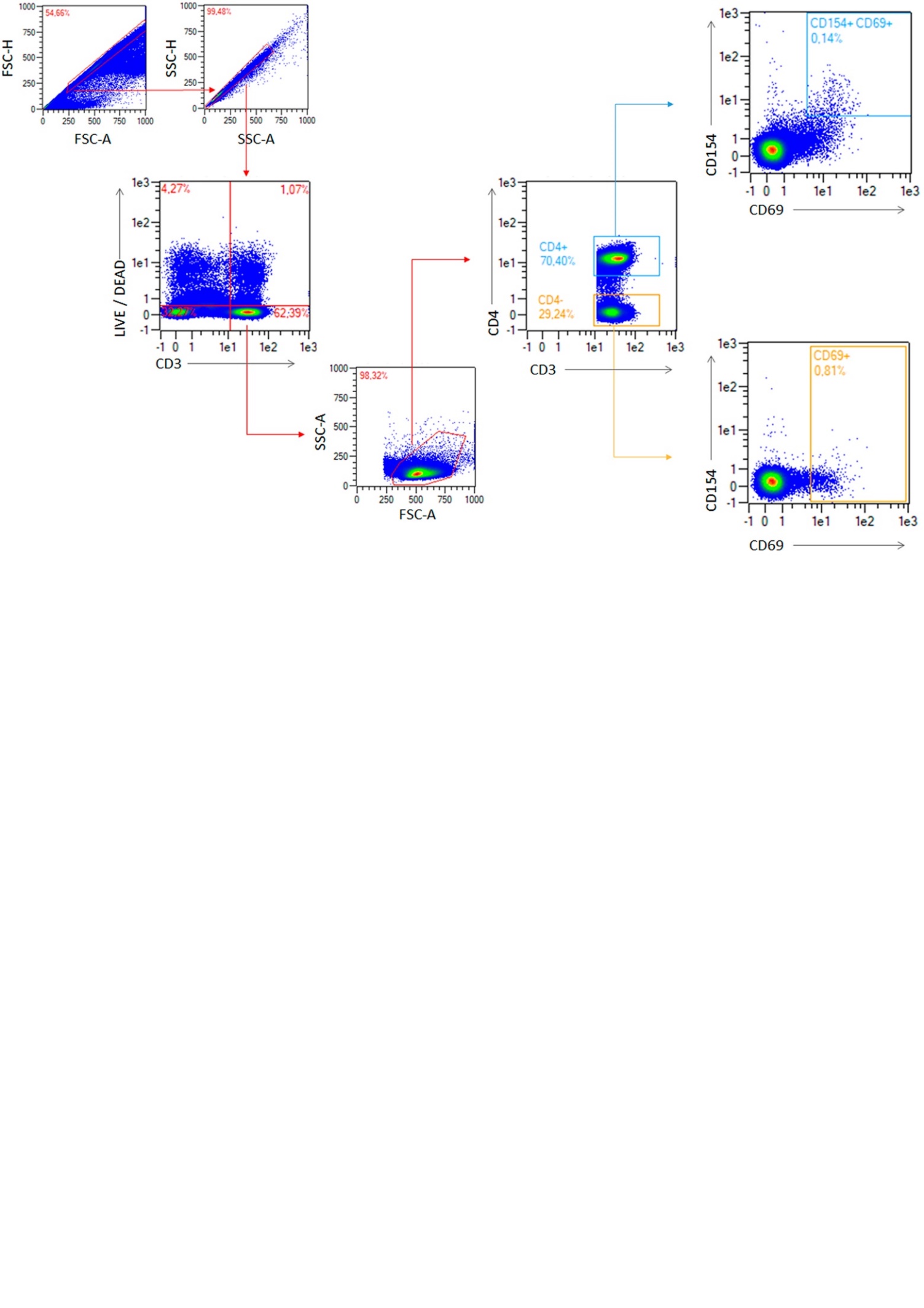
**

**Supplementary figure 3.** Flow cytometry gating of PBMCs for the specific response studies of T cells.
